# Single-Mode Tapered Vertical SU-8 Waveguide Fabricated by E-Beam Lithography for Analyte Sensing

**DOI:** 10.3390/s19153383

**Published:** 2019-08-01

**Authors:** Yu Xin, Gregory Pandraud, Yongmeng Zhang, Paddy French

**Affiliations:** 1 State Key Laboratory of NBC Protection for Civilian, Beijing 102205, China; 2 EEMCS, Delft University of Technology, 2628 CD Delft, The Netherlands; 3 College of Intelligence Science and Technology, National University of Defense Technology, Changsha 410073, China

**Keywords:** SU-8 photoresist, vertical waveguide, single-mode, evanescent sensing, E-beam lithography, taper

## Abstract

In this paper, we propose a novel vertical SU-8 waveguide for evanescent analyte sensing. The waveguide is designed to possess a vertical and narrow structure to generate evanescent waves on both sides of the waveguide’s surface, aimed at increasing the sensitivity by enlarging the sensing areas. We performed simulations to monitor the influence of different parameters on the waveguide’s performance, including its height and width. E-beam lithography was used to fabricate the structure, as this one-step direct writing process enables easy, fast, and high-resolution fabrication. Furthermore, it reduces the sidewall roughness and decreases the induced scattering loss, which is a major source of waveguide loss. Couplers were added to improve the coupling efficiency and alignment tolerance, and will contribute to the feasibility of a plug-and-play optical system. Optical measurements show that the transmission loss is 1.03 ± 0.19 dB/cm. The absorption sensitivity was measured to be 4.8 dB per refractive index unit (dB/RIU) for saline solutions with various concentrations.

## 1. Introduction

Integrated optical waveguide systems have been extensively investigated since they were first introduced by Miller in 1969 [1]. They are widely used in sensors, modulators [2], optical scanners [3], optical switches [4], etc. In the sensing area, evanescent waveguides have been studied by many researchers and have shown potential as biomedical and chemical sensors [5]. Polymers are important materials for micro-opto-electro-mechanical system (MOEMS) applications and are commonly used in integrated optics due to their adjustable refractive indices, good mechanical properties, low cost, compatibility with semiconductor technology, etc. [6]. SU-8, as an epoxy photon-sensitive photoresist that allows patterning by photolithography, simplifies fabrication processes. By avoiding dry etching, sidewall roughness is reduced and scattering loss is lowered. SU-8 can be fabricated by direct writing lithography such as E-beam lithography, allowing maskless processes, low-cost prototyping, fast and submicron fabrication with high precision [7]. Moreover, it is very stable after polymerization and hard baking, which is important for sensing [8,9,10]. Optically, it is transparent above 400 nm and the refractive index of SU-8 is 1.57 at 1.3 μm [7,8] making it suitable as a waveguide core material. Another important characteristic is that the surface of SU-8 has a good biocompatibility and its surface can be functionalized with antibodies [11], broadening the application of this material to biomedical sensing [8], medical diagnostic application [12], etc. Considering the characteristics of SU-8, it was chosen as a waveguide core material in this work. In general, basic waveguide structures include ridge waveguide, rib waveguide, slot waveguide, and hollow waveguide [13]. Most SU-8 waveguides investigated thus far were designed based on traditional waveguide structures with low height–width ratio. There are structures that have a relatively large size [14] fitting for optical interconnect, with a suspended structure to avoid leakage into substrate [15], or designed into multilayers with a modified small refractive index difference for single mode propagation [16]. They either have a relatively complex fabrication process or not aimed for sensing. In this paper, we propose for the first time a vertical waveguide structure with SU-8 as the core material for single-mode evanescent sensing. It has a high-aspect ratio structure and thus can create evanescent waves on both left and right side surfaces for sensing, which can increase sensitivity. The proposed waveguide can be achieved by one-step lithography and greatly decreases fabrication time and guarantees sidewall surface quality.

## 2. Structure Design and Simulation

In slab waveguides, light is only confined in one direction, while in ridge waveguides, light is confined in two directions. In this paper, ridge waveguides are classified into two categories: horizontal waveguides and vertical waveguides. A horizontal waveguide has a cross section with a height–width ratio smaller than 1, while a vertical waveguide has a cross section with a height–width ratio larger than 1.

### 2.1. Structure Analysis

**Figure 1:**
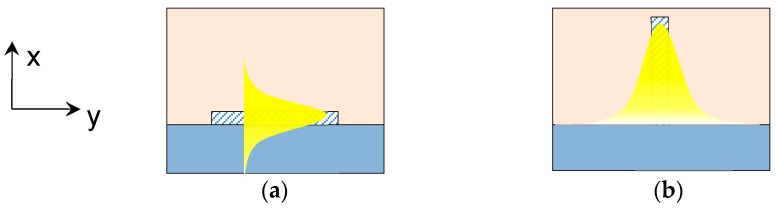
The evanescent wave distribution of: (**a**) horizontal waveguide and (**b**) vertical waveguide.

In horizontal waveguides, the optical ray is mainly bounced on the upper and lower surfaces and the evanescent wave is distributed on the upper cladding. Higher modes are mainly supported in the horizontal direction. While in vertical ones, the evanescent wave is distributed on the left and right sides of the waveguide and higher modes are supported in the vertical direction. The evanescent wave distributions in horizontal and vertical waveguides are shown in Figure 1a,b.

Sensitivity is an important criterion for waveguide-based sensing systems. It is defined as the amount of change in the sensor output resulting from a unit change on the sensor’s surface [17].

For evanescent wave sensing, the sensitivity depends on the optical energy distribution in the cladding area interacting with the measurand in the cladding when waveguide length is fixed. Therefore, achieving a waveguide with higher sensitivity depends on designing a structure that allows more optical energy to be distributed outside the waveguide core and reaching further into the cladding. Accordingly, the cladding filling factor in the cover medium, which is the proportion of the optical energy distributed in the cladding, can be used here as the criteria. The cladding filling factor $\Gamma_{c}$ is defined as [18,19,20]:

(1) $$\Gamma_{c}=\frac{\iint_{c}{\left|E(x,y)\right|}^{2}dxdy}{\iint_{\infty }{\left|E(x,y)\right|}^{2}dxdy},$$

where **E**(*x*,*y*) is the electric field vector and C is the cover medium area in the cladding.

Consequently, we define the ratio of cladding filling factor change to the refractive index change as optical sensitivity:

(2) $$S_{CFF}=\frac{\Delta \Gamma_{c}}{\Delta n_{c}}.\;$$

The absorption sensitivity here is defined as the ratio of absorption change to the refractive index change, as shown in the following equation:

(3) $$S_{A}=\frac{\Delta A}{\Delta n_{c}}\;,$$

where the output signal difference is the absorption change and the input signal variations is determined by the refractive index change. *A* is the evanescent absorption defined as:

(4) $$A=\log\left(\frac{I_{0}}{I_{a}}\right)=\alpha L\Gamma_{c},$$

where *I*_0_ is the light intensity transmitted when the cladding is not absorbing, *I_a_* is the light intensity transmitted when the cladding is absorbing, α is the absorption coefficient of the cladding, and *L* is the optical path length [21,22].

**Figure 2:**
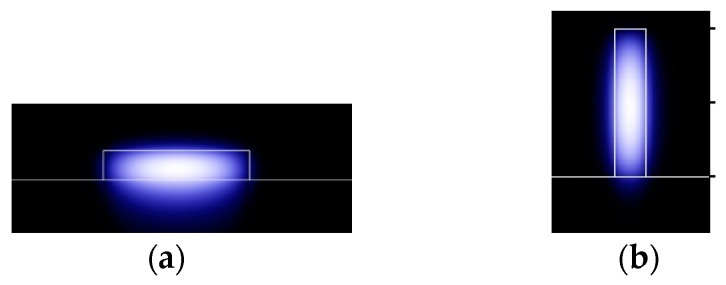
Fundamental mode in: (**a**) horizontal waveguide and (**b**) vertical waveguide.

Simulations were performed to study the cladding filling factor of horizontal and vertical silicon-on-insulator (SOI) waveguides to preliminarily compare the optical distribution. Two structures were set with reverse parameters, where the height of horizontal waveguide is the width of the vertical one, and vice versa. Results are shown in Table 1. In the horizontal SOI waveguide with *H* = 0.1 μm and *W* = 2 μm, the cladding filling factor in the left and right areas was less than 0.0015% for the fundamental TE_0,0_ mode, while that on the top was ~20%. In contrast, in a vertical SOI waveguide with *H* = 2 μm and *W* = 0.1 μm, the cladding filling factor in the left and right cladding was 46%, while that on the top was only 0.00127% for the fundamental TM_0,0_ mode. The fundamental mode in waveguides is shown in Figure 2. This comparison shows that the vertical waveguide not only has a larger sensing area, but also a higher cladding filling factor, which indicates a potential higher sensitivity. The vertical structure was therefore further studied for waveguide sensing.

**Figure 3:**
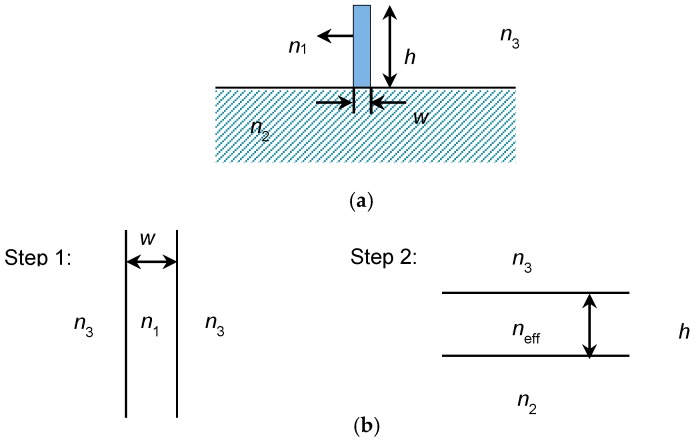
Effective index analysis method to analyze the vertical waveguide: (**a**) A vertical waveguide structure and (**b**) Solved by splitting into two planar.

The effective index analysis method was used to analyze and design the vertical SU-8 waveguide [20]. The vertical waveguide structure (shown in Figure 3a) can be considered as a combination of two imaginary planar waveguides as shown in Figure 3b. First, we solved the planar waveguide eigenvalue equations in one direction and then in the other direction. The effective index of the first imaginary waveguide was taken as the core refractive index of the second one. If the electric field is considered polarized in the *x* direction, which is transverse electric (TE) polarization, then the first decomposed vertical three-layer waveguide should be solved using transecerse magnetic (TM) eigenvalue equation. The three-layer planar waveguide is subsequently solved by the TE eigenvalue equation.

**Figure 4:**
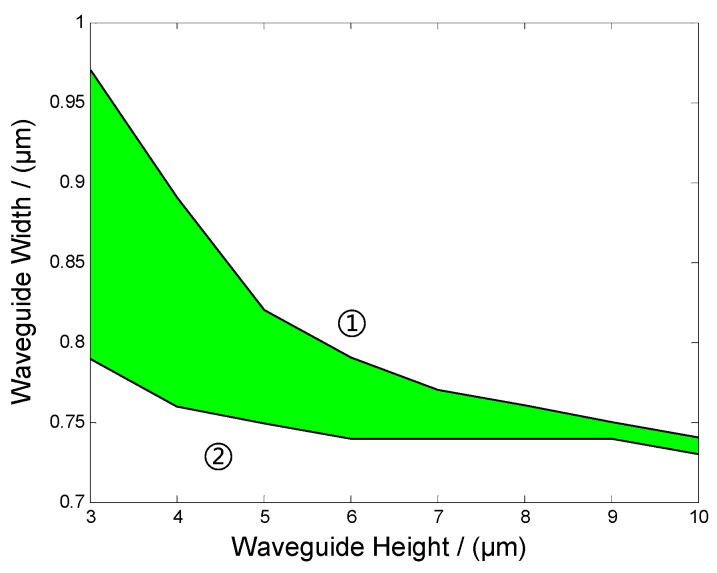
The single mode conditions of the vertical SU-8 waveguide.

In this context, the cutoff and single mode conditions can be calculated and used as design instructions. In this study, vertical SU-8 waveguides with different heights ranging from 3 µm to 10 µm were analyzed to obtain corresponding widths that meet the single mode condition. The results are shown in Figure 4. The green field between the two lines is where the parameters meet the single mode condition. Line 1 is the single mode line, above which, the structure will support multimode. While line 2 is the cutoff line, no mode will exist below in the cutoff region. These data show that with the increase in the height–width ratio, the single mode region becomes narrower and requirements for design and fabrication become stricter.

### 2.2. Simulations

**Figure 5:**
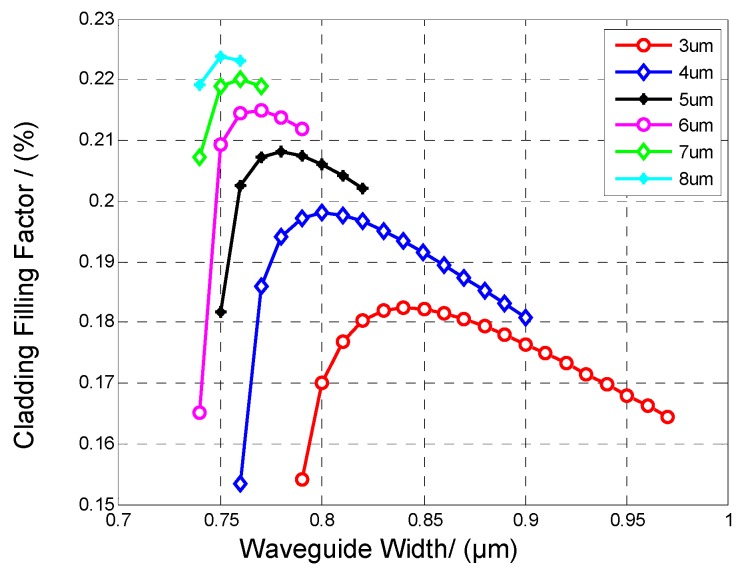
Cladding filling factors of waveguides with different heights and widths.

To maximize the cladding filling factor for a higher sensitivity, simulations were performed to obtain optimal parameters. Waveguide height and width both have an influence on the mode and cladding filling factor. For different heights, the cladding filling factor changes with width. Simulation results regarding the influence of waveguide width and height on cladding filling factors are shown in Figure 5. All the simulations were based on the waveguides with parameters meeting single mode conditions. The simulation results indicate that if the waveguide is higher, the waveguide can reach a larger cladding filling factor with the same width. However, it also requires a stricter tolerance for the waveguide width to meet a single mode condition, which translates into a tighter fabrication tolerance. Considering both the cladding filling factor and fabrication tolerance, 5 μm height and 0.8 μm width would be the optimal waveguide parameters.

**Figure 6:**
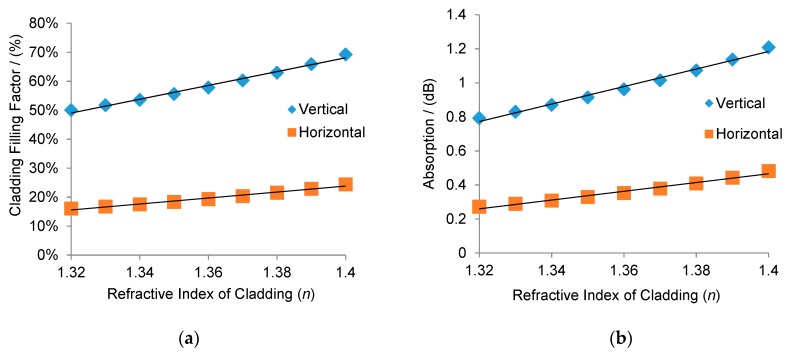
(**a**) Simulations on the optical sensitivity of horizontal and vertical waveguides; and (**b**) simulations on the absorption sensitivity of horizontal and vertical waveguides.

Simulations on the cladding filling factor with different cladding refractive indices were performed to compare the optical sensitivity between the designed waveguide and its corresponding horizontal one, with 5 µm width and 0.8 µm height. The results are plotted in Figure 6a, which shows that the cladding filling factor increased with an increase in the refractive index of the claddings. Moreover, the vertical SU-8 structure showed a higher sensitivity to the refractive index change in the cladding than the horizontal one. The sensitivity of the vertical one was 2.38 /RIU, while that of the horizontal one was only 1.03 /RIU. Subsequently, three-dimensional (3D) models developed from the designed structure were built to further simulate the absorption sensitivity of these two structures. The concentration of chemical solution will have an influence on both the real component (*n*) and the imaginary component (*k*) of its optical constant. Here, the simulations were conducted by changing the refractive index of the cladding while the absorption rate of the cladding is kept constant. It simulated the situation of saline sensing, where the refractive index changes with different saline concentrations and the absorption rate is dominated by water [23,24]. The sensing length was set as 1 cm. The simulation results are shown in Figure 6b. It can be calculated that the sensitivity of the vertical waveguide is 5.2 dB/RIU while that of the horizontal waveguide is 2.6 dB/RIU, demonstrating that the vertical waveguide has a higher sensitivity than the horizontal one. This result is in agreement with the cladding filling factor predication. These data prove that the cladding filling factor is an efficient indicator for estimating waveguide sensitivity.

## 3. Taper Design and Simulation

Coupling light into optical waveguides from an optical fiber is very important and critical as the size of the optical waveguide core is very small compared to the core of fiber. Direct coupling will always result in a big coupling loss and a difficult coupling operation, thus a coupler between the optical waveguide and optical source is needed to guarantee a reasonably good coupling efficiency and a relatively large alignment tolerance. There are various ways to couple light into the waveguide, where the common methods include end-fire coupling, prism coupling, grating coupling, and tapered coupling [20]. End-fire couplers are limited in the application because of strict alignment tolerance. In the case of prism couplers, it is difficult to find prism materials that meet the coupling conditions for most waveguides. The incident beam must be highly collimated and coupling efficiency is sensitive to the separation between the prism and the waveguide, so this coupling method is not practical. Grating coupling is achieved by introducing light at a specific angle to the grating part, and light will be coupled into the waveguide when the phase match condition is met. However, it has a theoretical low coupling efficiency and is not sufficiently robust for commercial device. Tapered coupling is an end-butt coupling, where light is first coupled to the thicker and wider taper part. Then, light is reflected to the narrower transfer part and gradually into the waveguide film or structure. The wider and higher tapered structure leads to an increase in the alignment tolerance. It is also easier to fabricate and works reasonably well [25]. 

**Figure 7:**
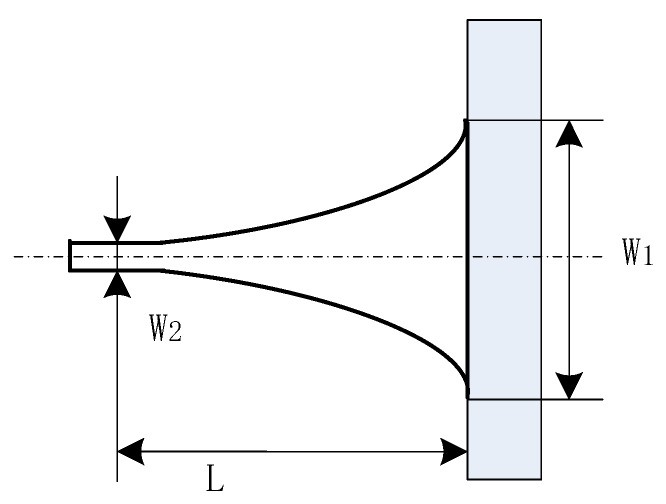
Taper schematic.

**Figure 8:**
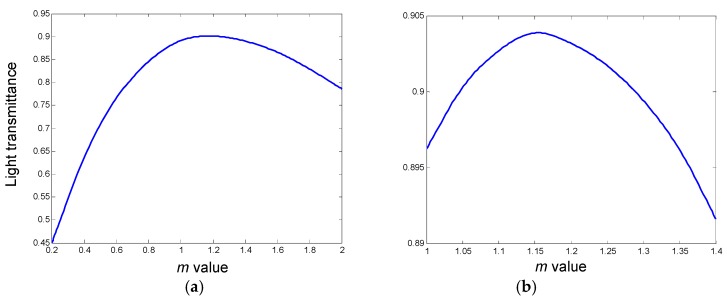
Transmission rate with different *m* values. (**a**) Sweep from 0.2 to 2; and (**b**) sweep around the peak value from 1 to 1.4.

The taper model was built as shown in Figure 7. The end of the waveguide is tapered horizontally to have a large coupling entrance, which contributes to increased coupling efficiency and alignment tolerance. To investigate how the taper profile influences coupling efficiency, an exponent function was used to define the profile of the taper as shown in eq 5 [26]. In this equation, *w*(*x*) is the taper width at the *x* axis and *L* is the total taper length; *w*(0) is taper width at the entrance with the value of *w*_1_; *w*(*L*) is the taper width of taper end, defined here as *w*_2_; and *m* is the parameter of the exponent function which defines the taper profile. To optimize the value of *m*, a parameter sweep from 0.2 to 2 was built up to track the light transmission rate through the waveguide. The simulation results plotted in Figure 8a show that for the exponent function defined taper, the transmission changes quite significantly with *m*. There exists an optimal parameter that enables the taper with the highest transmission rate. A more detailed sweep around the peak value from 1 to 1.4 was conducted to find the optimal value and the result was plotted in Figure 8b. The data show that when *m* = 1.15, the coupler is found to be the most efficient. When *m* = 1, the coupling efficiency is just slightly lower than the highest point but this would simplify our design, so *m* = 1 was chosen for the design.

(5) $$\{\begin{array}{l} w\left(x\right)=\alpha {\left(L-x\right)}^{m}+w_{2}\\ w\left(0\right)=w_{1}\\ w\left(L\right)=w_{2}\\ \alpha =\left(w_{1}-w_{2}\right)/L^{m} \end{array}.$$

**Figure 9:**
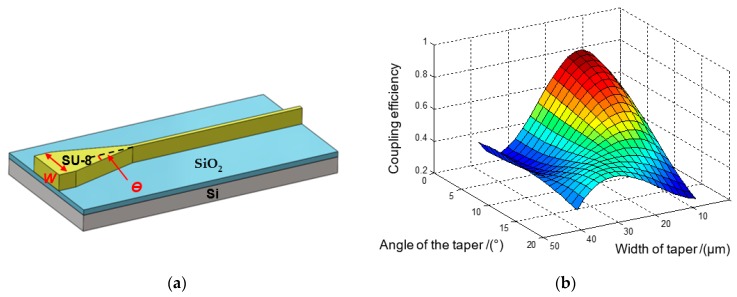
(**a**) Schematic of SU-8 waveguide with taper; and (**b**) the coupling efficiency of SU-8 taper coupler with different parameters.

The height of the SU-8 vertical waveguide is already relatively large in the vertical direction. Therefore, the added taper can increase the coupling efficiency and alignment tolerance in the horizontal direction and guarantee a proper performance in the vertical direction simultaneously. The schematic of a designed waveguide with taper is shown in Figure 9a. Taper width (*W*) and angle (*θ*) are two dominant factors influencing the coupling efficiency and alignment tolerance. Different taper widths and angles were combined in the simulation to obtain an optimal transmittance. The results are shown in Figure 9b. It can be concluded from the figure that an optimal parameter for the tapers exists. Generally, the coupling efficiency will become higher as the angle decreases. However, in a practical situation, when the angle is very small, the taper length will be very long, resulting in a big scattering loss induced by surface roughness. It also harms the compactness of the system. Therefore, the taper angle should be moderate. Meanwhile, when the taper’s length is fixed, alignment tolerance increases as the width increases, but the coupling efficiency decreases simultaneously. Taking all these factors into consideration, the taper parameters were chosen to be 20 μm wide with an approximately 5° angle.

## 4. Fabrication

**Figure 10:**
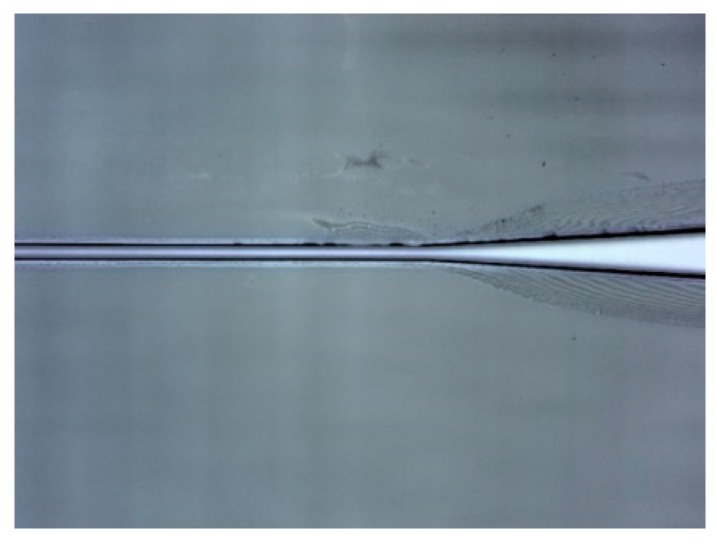
SU-8 optical waveguide with taper.

The waveguide fabrication was conducted at the Else Kooi Laboratory of TUD. A 3 µm-thick SiO_2_ was first thermally oxidized at 1100 °C on a silicon wafer as an isolation layer, acting as waveguide substrate and preventing light leakage into silicon underneath. Then a plasma treatment was conducted to improve the adhesion of SU-8 photoresist to the substrate. SU-8 3005 photoresist was then spin-coated onto the SiO_2_ layer to achieve a 5 µm thick with the following recipe: (1) spin at 500 rpm for 10 s with the acceleration of 100 rpm/sec and (2) spin at 3000 rpm for 30 s with acceleration of 300 rpm/sec [10]. Afterwards, the wafer was prebaked with slow temperature ramping up and cooling down to prevent any potential induced stress and guarantee good adhesion [27]. Then the whole wafer was processed by E-beam lithography using a Raith EBPG-5200. Post bake was also conducted with slow temperature ramping up and cooling down. Standard developing was continued to eliminate the uncrosslinked SU-8. Hard bake at 120 °C was performed consequently to stabilize the structure. Then the whole wafer was diced with a protection photoresist layer on top to prevent the structures from being damaged during the process. After dicing, the protecting resist layer was removed using acetone. The fabricated waveguides with tapers are shown in Figure 10. The structures attached well and remained intact on the substrate after dicing. 

**Figure 11:**
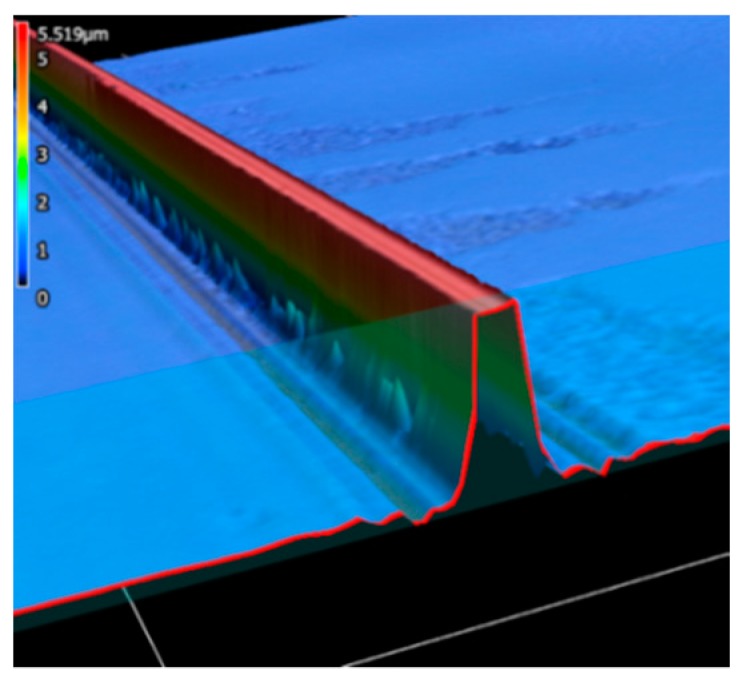
A 3D image of the cross section by Keyence.

**Figure 12:**
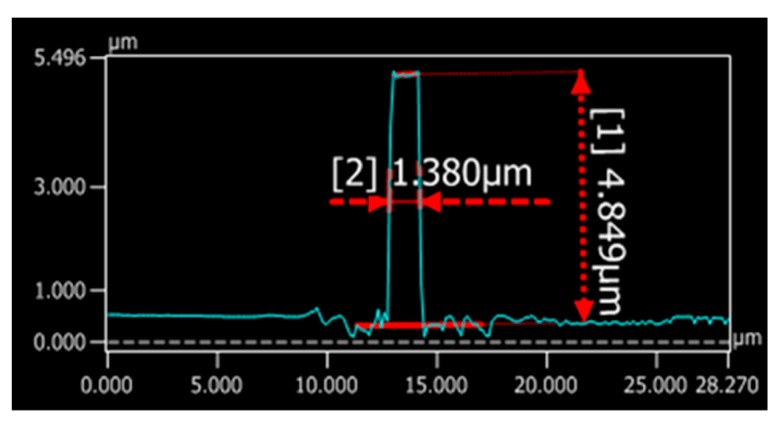
Waveguide height and width measurements.

Morphological measurements were taken under a Keyence VK-X 3D scanning confocal laser microscope. The 3D image in Figure 11 shows that the waveguide sensing part has a relatively vertical sidewall and reasonably good surface in the upper part. The cross section is shown in Figure 12 (1.38 µm wide and 4.849 µm high). The structures were slightly over exposed and the sidewall roughness was a little bit higher near the substrate with some residues, which can be improved by optimizing lithography parameters (expose dose, expose energy, and development time). The residues around the taper on the substrate can be better removed by increasing the developing time. From the sensing perspective, as most light is distributed in the middle area of the core, with a small amount near the contact surface of SU-8 and substrate, the residues will not have an obvious impact on the sensor’s performance. Moreover, the fact that our sensor detects measurand by comparing light intensity changes also makes the residue influence negligible.

## 5. Measurements

After fabrication, a series of tests was conducted with optical setups consisting of:

1. Waveguide with an adjustable platform;
2. Light source: Superluminescent Light-Emitting Diodes (SLEDs): EXS1320-2111, EXALOS), the SLEDs board driver, fibers (P3-SMF28-FC-2, Thorlabs);
3. Photodiode power sensors (Seri S122C, Thorlabs) and power meter (PM100D, Thorlab);
4. The optical source was connected using an FC connector with single mode fibers. The Fibers were mounted onto the 3-directional adjustable platform. The transmitted optical energy was detected by the photodiode and displayed on a power meter.

Propagation loss is a crucial specification of optical waveguides. Although it is not dominant in our application, it should be moderate not to diminish the optical energy applied for detection. The most common and simple way to measure optical waveguide propagation loss is the cut back method [20], which can be expressed as:

(6) $$\alpha =(\frac{1}{L_{1}-L_{2}})\ln(\frac{I_{1}}{I_{2}})$$

where *L*_1_ and *L*_2_ are waveguide lengths, and *I*_1_ and *I*_2_ are the transmitted light intensity. In this case, *L*_1_ is assumed larger than *L*_2_. Measurement accuracy can be improved by multiple measurements. Loss can be calculated by plotting optical loss against waveguide length. 

**Figure 13:**
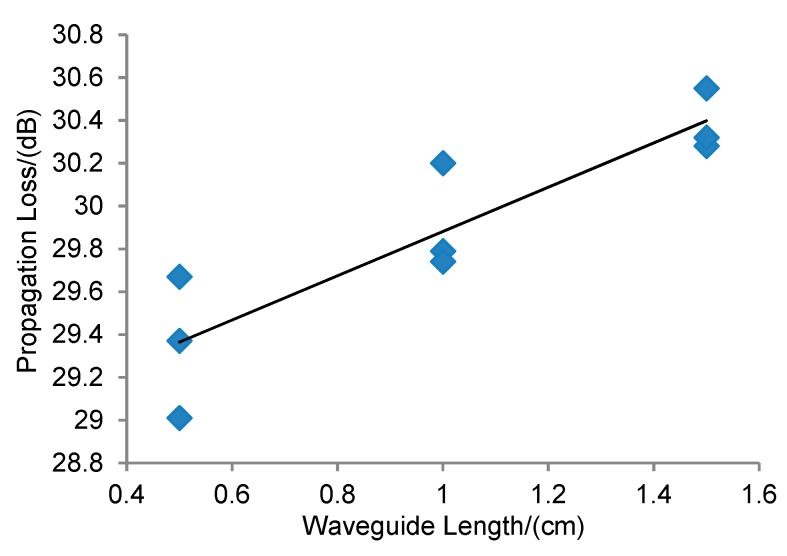
Cut back method to estimate the propagation loss in the SU-8 waveguide.

Waveguides with different lengths (0.5 cm, 1 cm, and 1.5 cm) were fabricated. After fabrication, optical measurements were conducted to obtain the propagation loss of the waveguide with different lengths. Results are shown in Figure 13. The propagation loss was calculated to be 1.03 ± 0.19 dB/cm for the designed SU-8 waveguide. The insertion loss was as big as 14 dB/facet as the samples were diced, and this mechanical process causes large surface roughness on the cross section. This can be optimized by cleaving the samples instead of dicing or by polishing the samples.

**Figure 14:**
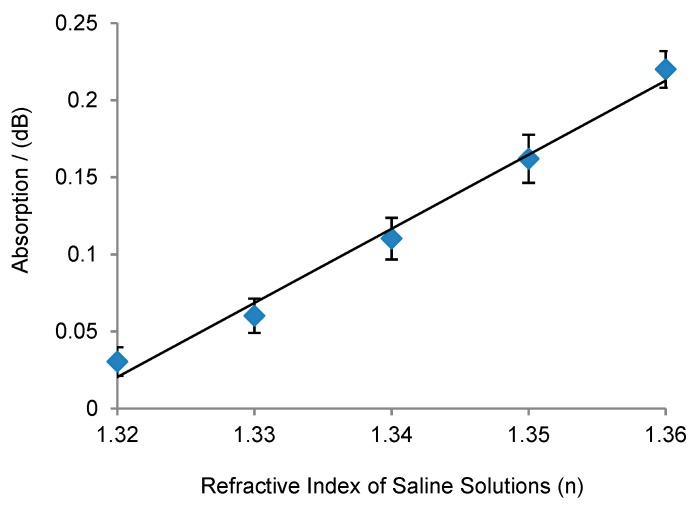
SU-8 waveguide sensitivity measurement with different saline concentrations.

Sensitivity experiments were performed using saline solutions with different concentrations. Different concentrations have different refractive indices. The refractive index changes with concentrations, and can be referred to in the study by Li et al. [24]. Absorption rate is dominated by water, so the influence of saline concentration on the cladding absorption rate can be neglected. However, changes in the refractive index will influence the cladding filling factor and optical distribution, which will influence the waveguide’s total absorption. Waveguide simulations were performed in COMSOL as shown in Figure 6b, and the sensitivity was calculated to be 5.2 dB/RIU. Measurements were carried on and the results are shown in Figure 14, demonstrating an increased absorption rate with an increase in the refractive index of saline solution. Absorption was plotted against the refractive index. The sensitivity was calculated to be to be 4.8 dB/RIU. The measured results are in agreement with the simulation prediction. The measured sensitivity is slightly lower than the theoretical value due to the fabrication errors. 

## 6. Discussions and Conclusions

In this paper, we put forward and fabricated an SU-8 waveguide with a novel vertical structure. With this design, the sensing area and the applicable evanescent wave were both increased and the waveguide sensitivity was improved compared to traditional horizontal waveguide structures. The simplicity of the proposed one-step maskless fabrication is very efficient at cutting down the design and the fabrication times. It also improves the quality of the waveguide’s sensing surface by avoiding the dry etching process. Optical measurements and sensitivity tests with different saline solutions demonstrate the feasibility and efficiency of the waveguide’s system. The test results are in agreement with the simulations and show improvements compared to the horizontal design. Tapers were added to increase the alignment tolerance and coupling efficiency for a plug-and-play system. As SU-8 is biocompatible and can be functionalized, further biomedical tests can be performed to broaden the system’s applicability in the biomedical field.

## Figures and Tables

**Table 1:** Comparison of energy distribution of horizontal and vertical structures.

	Width	Height	Cladding Filling Factor (On Top)	Cladding Filling Factor (On Sides)
Horizontal	2 µm	0.1 µm	20%	0.0015%
Vertical	0.1 µm	2 µm	0.00127%	46%
